# Evidence of Eczematous Skin Characteristics in Mild Allergic Asthma

**DOI:** 10.1002/clt2.70091

**Published:** 2025-08-06

**Authors:** Anchalee Senavonge, Ruth P. Cusack, Christiane E. Whetstone, Nadia Alsaji, Karen J. Howie, Caitlin Stevens, Jennifer Wattie, Lesley Wiltshire, Roma Sehmi, Paul M. O'Byrne, Hermenio Lima, Gail M. Gauvreau

**Affiliations:** ^1^ Division of Allergy, Immunology and Rheumatology Department of Pediatrics Faculty of Medicine Chulalongkorn University and BNH Hospital Bangkok Thailand; ^2^ Division of Respirology Department of Medicine Faculty of Health Sciences McMaster University Hamilton Ontario Canada; ^3^ Division of Allergy and Clinical Immunology Department of Medicine, Faculty of Health Sciences, McMaster University Hamilton Ontario Canada

To the Editor,

Atopic dermatitis (AD) and asthma are associated through common inflammatory pathways, immunologic crosstalk, barrier disruption, and genetic predisposition [1]. Approximately 50%–70% of asthmatic patients have AD, while 20%–40% of AD patients are diagnosed with asthma [2, 3]. Medications that inhibit type 2 inflammation, such as corticosteroids and dupilumab, effectively treat both conditions, suggesting an overlap in the pathogenesis of AD and asthma. Since there have been no objective skin assessments in asthmatics without a history or clinical findings of AD, we aimed to identify the proportion and characteristics of asthmatics who display skin features consistent with those of AD. The Hamilton Integrated Research Ethics Board approved this study.

Mild allergic asthmatics without a historic or current clinical findings of AD were recruited. Participants had not used any anti‐inflammatory therapy for treatment of asthma, including but not limited to corticosteroids or biologics, for over 1 month before enrollment. They were characterized as asthmatic by positive methacholine challenge (PC_20_ ≤ 16 mg/mL), and their allergic sensitization was documented through a positive skin prick test to a panel of aeroallergens. Measurements of blood total immunoglobulin E (IgE) and blood and sputum eosinophil counts were conducted. Skin biopsies of unaffected skin were obtained from the lower back using a 4 mm punch to evaluate spongiosis and vacuolization of the dermo‐epidermal junction, which are characteristic findings in the skin of AD patients. Epidermal spongiosis and dermal neutrophilic and lymphocytic infiltration were measured semi‐quantitatively using a 4‐point scale. The severity of spongiosis was assessed as 0 (absence of haloing/vacuoles or cellular infiltrate), 1 (haloing present), 2 (haloing present with vacuoles formed), or 3 (numerous vacuoles with intravacuolar lymphocytic or neutrophilic infiltrate) [4]. Participants were then separated into groups based on the absence or presence of epidermal spongiosis, defined as a score of ≥ 2. Groups were compared using the independent samples *t*‐test for normally‐distributed data, the Mann–Whitney *U* test for non‐normally distributed data, and Fisher's Exact Test for categorical data, and summary statistics are presented as mean (± SD), median (range), and number (%), respectively. The threshold for statistical significance was set at *p* < 0.05.

Fourteen asthmatic participants completed the study. The geometric mean (range) methacholine PC_20_ was 4.5 (0.48–13.8) mg/mL, with an FEV_1_ of 100 ± 12.7% predicted and an FEV1/FVC ratio of 0.83 ± 0.06. Allergic rhinitis was reported in 11 out of 14 (78.5%) participants. Four of the 14 participants (28.6%) had epidermal spongiosis and vacuolar infiltration consistent with eczematous skin of AD (Table 1). Low dermal lymphocyte infiltration was observed in three participants in the non‐eczematous group, and none of the biopsies showed signs of neutrophilic infiltration. When comparing participants with and without eczematous biopsies, we found that the eczematous group was significantly older at the time of biopsy collection (35.7 ± 12 vs. 25.6 ± 5.6 years of age), with a younger age at asthma onset (6.25 ± 2.8 vs. 15.8 ± 8.3 years of age). They also exhibited a larger skin response to house dust mite: (*Dermatophagoides pteronyssinus* 5.5 [3.75–7.63] vs. 2 [0–3] mm diameter wheal and *Dermatophagoides farinae* 5.25 [3.75–6.63] vs. 1 [0–3.75] mm diameter wheal; *p* < 0.05). The size of skin wheals to cat, dog, horse, feather, mold, dictyopteran, Alternaria, Aspergillus, weed, ragweed, tree, or grass pollen was similar between the two groups. Blood total IgE levels were numerically higher in the eczematous biopsy group (443.5 ± 634 vs. 261.1 ± 397 IU/mL, *p* = 0.28). Methacholine PC_20_, spirometry, and sputum eosinophil levels did not differ between the two groups. Studies comparing early‐onset asthma with late‐onset asthma have revealed different phenotypes with distinct clinical characteristics and comorbidities. In early‐onset asthma, atopic comorbidities, such as allergic rhinitis, and type 2 inflammatory patterns, as indicated by elevated serum IgE and blood eosinophils, are more prevalent. In contrast, late‐onset asthma is prone to comorbid obesity [5]. In this population of mild allergic asthmatics, we found no connection between AD and indices of asthma severity, as measured by methacholine and airway eosinophils. This could be due to the limited number of participants available for biopsy or the narrow range of asthma severity (all mild asthma) included in this study.

**TABLE 1:** Clinical characteristics of asthma patients with versus without eczematous changes on skin biopsy.

Clinical characteristics	Eczematous skin *N* = 4	Normal skin *N* = 10	*p* value
Skin			
Epidermal spongiosis score	2 (1.5–2)	0 (0–0)	—
Lymphocytic dermis score	0 (0–0)	0 (0–1)	0.5
Neutrophilic dermis score	0 (0–0)	0 (0–0)	> 0.99
Age (years)	35.7 ± 12	25.6 ± 5.6	0.03*
Male sex	2/4 (50%)	9/10 (90%)	0.18
Race			
Caucasian	4/4 (100%)	7/10 (70%)	> 0.99
Asian	0	1/10 (10%)	
Black	0	1/10 (10%)	
Hispanic	0	1/10 (10%)	
Age of asthma onset (years)	6.25 ± 2.8	15.8 ± 8.3	0.03*
Other allergic conditions			
Allergic rhinitis	3/4 (75%)	8/10 (80%)	0.43
Food/Drug allergy	0	0	—
Skin test wheal frequency; diameter (mm)^a^			
*Dermatophagoides pteronyssinus*	4/4 (100%); 5.5 (3.75–7.63)	6/10 (60%); 2 (0–3)	0.25; 0.008*
*Dermatophagoides farinae*	4/4 (100%); 5.25 (3.75–6.63)	5/10 (50%); 1 (0–3.75)	0.22; 0.045*
Cat	2/4 (50%); 1 (0–3)	6/10 (60%); 2.5 (0–5.63)	> 0.99; 0.64
Dog	2/4 (50%); 1 (0–2.5)	4/10 (40%); 0 (0–2)	> 0.99; 0.66
Horse	2/4 (50%); 1.5 (0–4.75)	5/10 (50%); 1 (0–5.88)	> 0.99; > 0.99
Feather	0/4 (0%) 0 (0–0)	2/10 (20%); 0 (0–0)	> 0.99; 0.56
Alternaria	2/4 (50%); 1 (0–2)	5/10 (50%); 1 (0–3.75)	> 0.99; 0.64
Hormodendrum	2/4 (50%); 1 (0–2.25)	3/10 (30%); 0 (0–1.5)	0.58; 0.36
Aspergillosus	1/4 (25%); 0 (0–0.5)	2/10 (20%); 0 (0–0)	> 0.99; > 0.99
Grass	3/4 (75%); 2.5 (1.5–4.36)	6/10 (60%); 3.25 (0–4.5)	> 0.99; 0.48
Tree mix	1/4 (25%); 0 (0–0.5)	5/10 (50%); 1 (0–3)	0.58; 0.33
Ragweed	2/4 (50%); 1 (0–3.13)	5/10 (50%); 1.75 (0–5)	> 0.99; 0.78
Weed mix (no ragweed)	1/4 (25%); 0 (0–1)	3/10 (30%); 0 (0–2.25)	> 0.99; 0.76
Blood cell count (cells/μL)			
Eosinophils	100 (100–125)	200 (100–275)	0.48
Lymphocytes	1425 ± 376	2000 ± 404	0.02*
Basophils	0 (0–0)	50 (0–100)	0.04*
Neutrophils	4630 ± 1696	3850 ± 1845	0.25
Monocytes	450 (400–500)	400 (400–650)	0.99
Total IgE (IU/mL)	113.5 (76.75–480.25)	83.5 (28.75–185.75)	0.64
Methacholine PC_20_ (mg/mL)	5.5 (2.2, 13.8)	4.2 (0.5, 11.7)	0.35
Spirometry			
FVC%	105.3 ± 9.3	101.2 ± 12.1	0.3
FEV1%	104.3 ± 12.1	98.4 ± 12.6	0.24
FEV1/FVC	0.81 ± 0.09	0.84 ± 0.04	0.29
Sputum eosinophils	0.45 (0.21–0.75)	2.45 (0.38–5.15)	0.35

*Note:* Data are presented as No. (%), mean ± SD, median (range). Methacholine PC_20_ is presented as geometric mean (range).

Abbreviations: — = statistical test not performed, FEV_1_ = Forced expiratory volume in one second, FVC = Forced vital capacity, IgE = Immunoglobulin E, PC_20_ = provocative concentration of methacholine causing a decrease of 20% in the FEV_1_.

^a^ Includes data from all patients (positive and negative tests).

*p value < 0.05 not corrected for multiple comparisons.

In conclusion, the current study underscores the distinct early‐onset asthma phenotype of participants in this study and the high frequency of atopic comorbidity of allergic rhinitis. Of this phenotype, those with pathological signs of eczematous skin had a significantly earlier age of asthma onset. Notably, in other studies, older age has been associated with greater changes in dermatitis symptoms following HDM exposure, which could result from longer sensitization to the allergen, as Th2 biomarkers are related to these two comorbidities [2, 3]. Genetic predisposition or immune signaling via the Th2 pathway could be an underlying explanation. HDM sensitization has been observed in both AD and asthma. Mite allergen exhibits protease activity that can influence the permeability of the respiratory and skin epidermal barriers through the activation of protease‐activated receptor 2 (PAR2). Previous studies have demonstrated that AD severity is associated with HDM concentrations [6, 7]. Moreover, HDM immunotherapy demonstrates clinical effectiveness in both asthma and AD, suggesting that dust mites can act as a significant trigger through IgE‐mediated mechanisms [8, 9]. A larger study with follow‐up is needed to fully understand the relationship between skin and airway inflammation in allergic patients. Through the evaluation of skin biopsies in asthmatic participants with no history of AD, this study has confirmed that early‐onset asthma patients with sensitization to house dust mite should be closely monitored for the development of atopic dermatitis.

## Author Contributions

**Anchalee Senavonge:** writing – review and editing, writing – original draft, formal analysis. **Ruth P. Cusack:** conceptualization, methodology, data curation, project administration, investigation. **Christiane E. Whetstone:** writing – review and editing, formal analysis, data curation, conceptualization, methodology, project administration. **Nadia Alsaji:** data curation, methodology. **Karen J. Howie:** project administration. **Caitlin Stevens:** project administration. **Jennifer Wattie:** data curation. **Lesley Wiltshire:** data curation. **Roma Sehmi:** supervision. **Paul M. O’Byrne:** supervision. **Hermenio Lima:** conceptualization, investigation, supervision, methodology, writing – review and editing. **Gail M. Gauvreau:** conceptualization, methodology, data curation, supervision, writing – review and editing, investigation, formal analysis, writing – original draft.

## Conflicts of Interest

R.P.C., A.S., C.E.W., N.A., K.J.H., C.S., J.W., L.W., R.S., P.M.O., G.M.G. declares have no conflict of interest. H. Lima has received consulting fees, attended advisory boards, and participated in clinical trials for the following: Aslam Pharm, AbbVie (Abbott), Amgen, AstraZeneca, Bausch Health, Bristol‐Myers‐Squibb, Celgene, Dermira, Eli Lilly, GSK, Incyte, Janssen, La Roche‐Posay, Leo Pharmaceutics, Merck Sharp & Dohme, Moonlake, Novartis, Pediapharma, Pfizer, RAPT, Regeneron, Sanofi, Takeda, and UBS. G.M.G. has received consulting fees and participated in clinical trials for the following: AstraZeneca, Genentech, Third Harmonics Bio, Blueprint Medicines, Biohaven, Jasper Therapeutics

## Data Availability

The authors have nothing to report.
